# Heterogeneity of dengue transmission in an endemic area of Colombia

**DOI:** 10.1371/journal.pntd.0008122

**Published:** 2020-09-14

**Authors:** María Isabel Estupiñán Cárdenas, Víctor Mauricio Herrera, María Consuelo Miranda Montoya, Anyela Lozano Parra, Zuly Milena Zaraza Moncayo, Janeth Patricia Flórez García, Isabel Rodríguez Barraquer, Luis Ángel Villar Centeno

**Affiliations:** 1 Grupo de Epidemiología Clínica, Universidad Industrial de Santander, Bucaramanga, Santander, Colombia; 2 Division of HIV, ID and Global Medicine, Department of Medicine, University of California, San Francisco, San Francisco, California, United States of America; Institute for Disease Modeling, UNITED STATES

## Abstract

Population based serological surveys are the gold-standard to quantify dengue (DENV) transmission. The purpose of this study was to estimate the age-specific seroprevalence and the force of infection of DENV in an endemic area of Colombia. Between July and October 2014, we conducted a household based cross-sectional survey among 1.037 individuals aged 2 to 40 years living in 40 randomly selected locations in urban Piedecuesta, Santander, Colombia. In addition, we also enrolled 246 indviduals living in rural “veredas”. Participants were asked to answer a questionnaire that included demographic, socioeconomic and environmental questions and to provide a 5 ml blood sample. Sera were tested using the IgG indirect ELISA (Panbio) kit to determine past DENV infection. The overall DENV seroprevalence was 70% (95% CI = 67%-71%), but was significantly higher in urban (81%, 95% CI = 78%-83%) as compared to rural (21%, 95% CI = 17%-27%) locations. Age was a major predictor of seropositivity, consistent with endemic circulation of the virus. Using catalytic models we estimated that on average, 12% (95%CI = 11%-13%) of susceptible individuals living in the city are infected by DENV each year. Beyond age, the only predictor of seropositivity in urban locations was prior history of dengue diagnosed by a physician (aPR 1.15, 95% CI = 0.98–1.35). Among participants living in rural settings, those that reported traveling outside of their vereda were more likely to be seropositive (aPR 3.60, 95%CI = 1.54–8.42) as well as those who were born outside of Santander department (aPR = 2.77, 95%CI = 1.20–6.37). These results are consistent with long term endemic circulation of DENV in Piedecuesta, with large heterogeneities between urban and rural areas located just a few kilometers apart. Design of DENV control interventions, including vaccination, will need to consider this fine scale spatial heterogeneity.

## Author summary

Dengue is the most rapidly expanding arbovirus in the world. Counts of reported dengue cases, as usually reported by surveillance systems, are a poor metric of the underlying transmission because most of the infections are asymptomatic. Population based serological surveys, where the prevalence of antibodies to dengue virus are measured in a random sample of the population, are considered the gold-standard to quantify dengue transmission. In this study, the authors conducted a population based serological survey in urban and rural areas of Piedecuesta, Santander, Colombia. Seropositivity was significantly higher in urban as compared to rural locations. Age was a major predictor of seropositivity, consistent with long-term circulation of the virus in this setting. They estimated that, on average, 12% of susceptible individuals living in urban locations get infected by dengue each year. The only predictor of prior dengue infection in urban inhabitants was self-reported history dengue. In rural locations, traveling regularly outside of their “vereda” (neighborhood) and having been born outside of Santander department were associated with seropositivity.

These results show that the Piedecuesta municipality is an endemic area of dengue transmission, with large heterogeneities between urban and rural settings. Control interventions, including vaccination should be targeted at fine spatial scales.

## Introduction

Dengue virus (DENV) is the most rapidly spreading arboviral infection and causes an estimated 390 million infections and 60 million symptomatic cases each year across the globe[1–2]. It has also been estimated that approximately 40% of the world’s population lives in tropical and subtropical areas at risk of DENV transmission, amounting to 2500 million people in over 100 countries [3].

For diseases like dengue, where a large proportion of infections are asymptomatic or cause a mild febrile syndrome, it is not possible to quantify transmission using the incidence of clinically suspected cases, as usually reported by surveillance systems, as it only represents a fraction of infections[4–5]. Age-stratified seroprevalence studies, that directly measure the proportion of the population that has been infected, are the gold standard to estimate key epidemiological parameters including the force of infection. The importance of age-stratified serological surveys was underscored in July 2016, when the WHO recommended that only populations with high DENV transmission, defined as 70% or greater seroprevalence, should consider the introduction of the first licensed DENV vaccine (Dengvaxia)[6–7]. This recommendation was later modified and currently vaccination is only recommended in those individuals who have been infected by DENV in the past, as determined by an individual pre-vaccination screening. However, these recommendatios made evident the big gaps that exist in our knowledge of DENV transmission at national and subnational scales[8].

There is a need for high quality serological studies to inform where to target interventions, including vector-control and vaccination[9]. A recently published scoping review identified a single serosurvey conducted in Colombia during the last 10 years and it was in an area of low transmission. Furthermore, even though there has been much discussion about the re-emergence of DENV in South America, this review only identified three recent serosurveys from Brazil and one from Perú[10].

Here, we present the results of a household-based cross-sectional serosurvey in urban and rural areas of Piedecuesta, Colombia, an area known to be hyperendemic for DENV[11]. The purpose of the study was to estimate the age-specific seroprevalence and transmission intensity of DENV and to characterize risk factors for infection.

## Methods

### Study design

Between July and October 2014, we conducted a household-based cross-sectional survey among 1.037 individuals aged 2 to 40 years living in 40 probabilistically selected locations in urban area of Piedecuesta. In addition, we enrolled 246 individuals living in a rural “vereda” (neighborhood). Serum samples were obtained from all participants and tested for IgG against DENV virus.

### Study setting

Piedecuesta is one of the four municipalities of the Metropolitan Area of Bucaramanga (MAB), which is the most densely populated area of Santander department (state) in northeastern Colombia. It has a population of about 150.000 inhabitants of whom almost 20% live in rural areas. The urban center is located at an elevation of 1.005 m above sea level, but the elevation of rural neighborhoods (“veredas”) is highly variable and ranges between 600–3600 m [12]. The selected rural “vereda” is located at 2.149 m above sea level and is about 10 km from the urban center.

DENV has been known to be endemic in Santander for at least 19 years and the four serotypes of the virus have been isolated [13]. According to the Instituto Nacional de Salud (Colombian National Institute of Health), the dengue incidence rate in Santander between 2013 to 2016 on average was 681 cases per 100.000 inhabitants and Piedecuesta is consistently among the top 10 municipalities that report the highest incidence of cases [14].

### Selection of blocks and households

We obtained a probabilistic sample of forty blocks within the town of Piedecuesta with probability proportional to population size. The information on the demographics of the municipality was provided by the National Administrative Department of Statistics (DANE), the entity in charge of planning, surveying, analyzing and disseminating local statistics in Colombia [15]. Furthermore, we selected a rural “vereda” for additional sampling. The selection of the rural “vereda” was non-probabilistic, and based on accessibility for study-team members.

For each selected block within the urban area, the study-team leader randomly selected a corner of the block as a starting point to conduct the visit of houses. Houses in the block were visited and invited to participate in a clockwise direction. Since the rural “vereda” spanned a much larger area, 8 random starting points were selected to approach households.

### Participants

Individuals living in the selected households who were between 2 and 40 years of age were invited to participate in the study and to sign the informed consent. Exclusion criteria included health conditions that contraindicated blood sample collection and mental or physical disabilities that limited the ability to give consent.

### Study procedures

All participants were asked to provide a 5 ml venous blood sample and to answer a brief questionnaire that included demographic information, data on daily activities and past history of dengue. Additionally, the head of each household was asked to answer a questionnaire about household characteristics.

Blood samples were collected by trained phlebotomist in tubes containing no anticoagulants and stored in portable coolers with a certified temperature control system. At the end of each day, the samples were transported to the AEDES Network Laboratory (Universidad Industrial de Santander) where they were centrifuged and aliquoted within six hours of collection. All the aliquots were stored at -80°C until the serological testing was completed.

Prior DENV infection was determined using the Panbio Dengue IgG Indirect ELISA (Inverness Medical Innovations, Brisbane, Australia). All serological testing was conducted at the AEDES Network Laboratory in Bucaramanga, following the manufacturer’s instructions.

### Statistical analyses

#### Analyses

The general characteristics of the sample were described using absolute frequencies, relative frequencies, medians and interquartile ranges (IQR). Contrast between rural and urban areas were done using chi squared and Mann Whitney tests.

Poisson mixed-effects models were used to explore potential risk factors for DENV infection. Models included random effects at the block level to account for clustering. Data from urban and rural areas were analyzed separately as descriptive analyses suggested very different patterns. Those variables with p value <0.25 in the null models were taken into account for the adjusted regression models. Then the final models were selected based on Akaike’s Information Criterion (AIC).

To quantify transmission intensity of DENV in Piedecuesta, we used the age-specific seroprevalence data to estimate the force of infection (FOI, the rate at which susceptible individuals get infected) and basic reproductive number as previously described[16–17].

All analyses were conducted in Stata v12 and in R statistical software[18–19]. Data for the main analyses is available in the supplementary material.

#### Sample size calculation

A sample size of 150 participants per age-group (2–5 years, 6–10 years, 11–15 years, 16–20 years, 21–25 years, 26–30 years, 31–35 years, 36–40 years) was estimated to achieve a minimal precision of 0.1 (α = 0.05) in each age-specific estimate. This estimate assumed a conservative seroprevalence of 50% and a design effect of 2 and implied a total sample size of approximately 1200 individuals (1000 from the urban area and 200 from the rural area).

### Ethical Review

This study was approved by the Institutional Review Boards at Universidad Industrial de Santander (Minute No. 20/Dec 19^th^ of 2013). All adult participants provided written consent after receiving a detailed explanation of the study and procedures. Children from 7 to 17 years of age provided their assent to participate, and parents or guardians of all child participants were asked to provide written consent on their behalf. Also adult participants provided written consent after a full explanation of the study was provided. All data were handled confidentially and anonymously.

## Results

**Fig 1:**
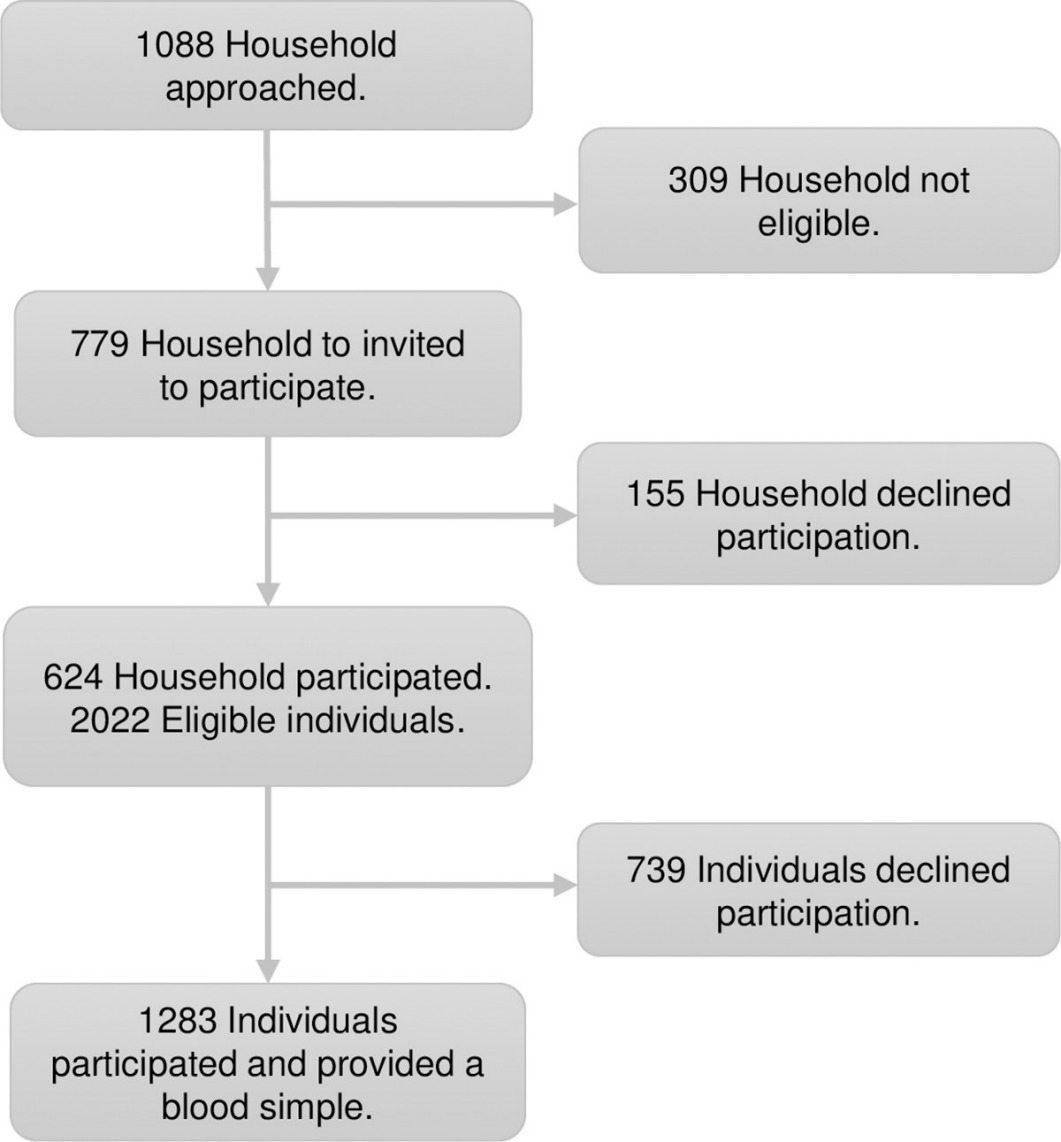
Flowchart of the inclusion of households and participants.

**Fig 2:**
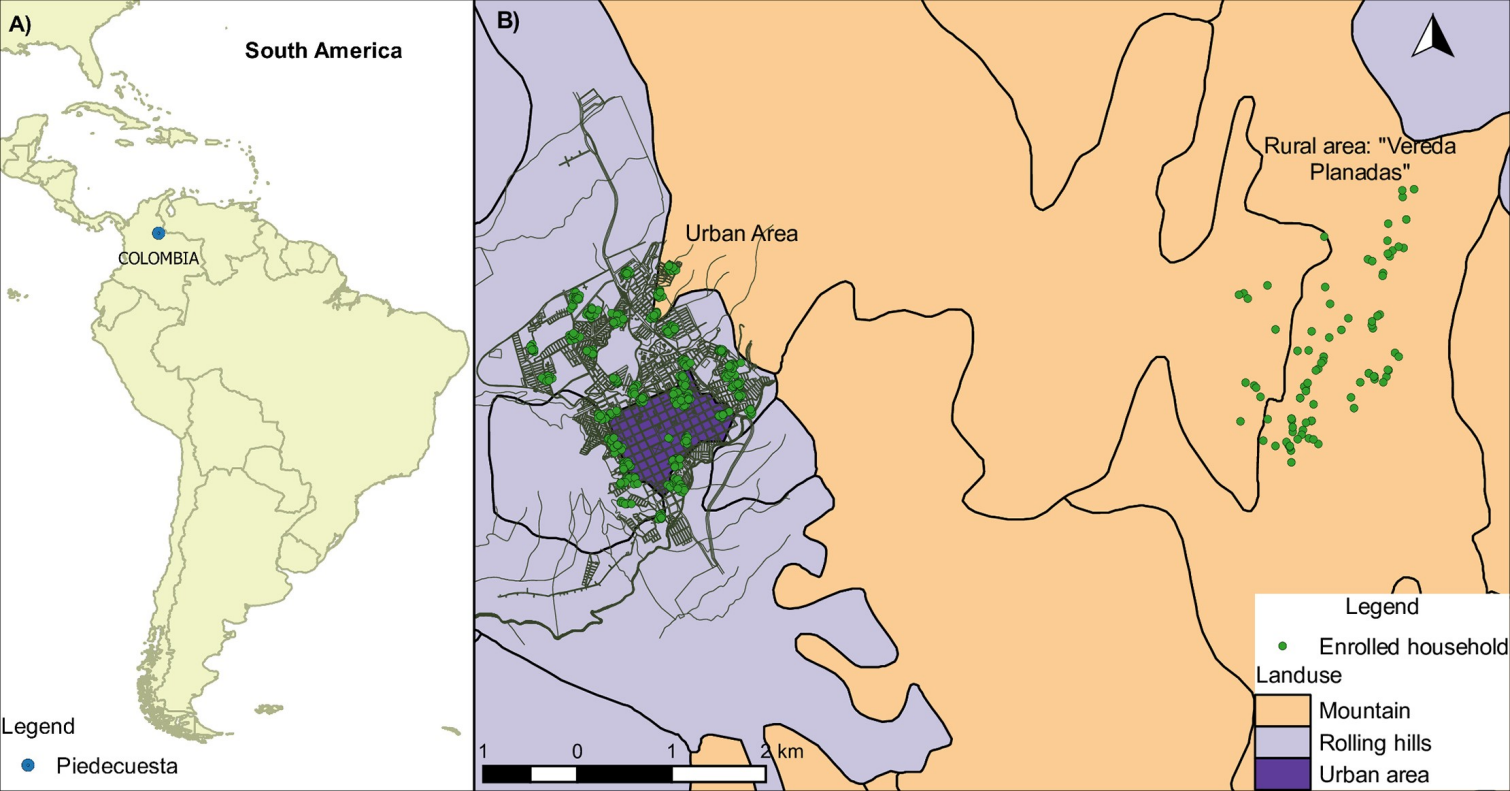
A. Location of Piedecuesta in Colombia. B. Location of enrolled households in Piedecuesta.

Between July and October 2014 we enrolled 1.283 participants living in 624 households located in 40 blocks in the urban area of Piedecuesta and 8 locations in the rural “vereda”. Household and individual participation rates were 80% and 64% respectively (Fig 1), but ranged between 50% and 100% in different locations. Fig 2 shows the locations of the households in the urban and rural areas.

### Characteristics of participants and households

The mean age of the participants was 20.7 years (95% CI = 20.1–21.3) and 758/1.283 (59%) were female. While the majority of participants were aware of dengue as a main health issue (1.058/1.283, 82%) only 229 (17%) reported having had dengue in the past (Table 1.). The proportion of IgG antibodies (previous infection of dengue) was higher in urban (218/1.037, 21%) as compared to rural areas 11/246, 4%, p-value = 0.001).

**Table 1:** Main characteristics of participants from urban and rural areas. Piedecuesta, 2014.

Characteristics	Urban. n (%)	Rural. n (%)	Overall. n (%)
**Number of individuals**	1.037	246	1.283
**Age (in years)** *2–5* *6–15* *16–25* *26–40*	103(10) 250(24) 306(29) 378(36)	24(10) 77(31) 50(20) 95(39)	127(10) 327(25) 356(28) 473(37)
**Female**	623 (60)	135 (55)	758 (59)
**Occupation** *Student* *Independent* *Homemaker* *Employee* *Unemployed*	445 (43) 196 (19) 158 (15) 154 (15) 20 (2)	90 (36) 85 (34) 49 (20) 5 (2) 1 (0.4)	535 (41) 281(22) 207 (16) 159 (12) 21 (2)
**Education** *None* *Preschool* *Primary* *Secondary* *Technical* *Undergraduate* *Postgraduate*	54 (5) 47 (4) 205 (20) 454 (44) 138 (13) 124 (12) 15 (1)	15 (6) 8 (3) 136 (55) 82 (33) 3 (1) 0 (0) 0 (0)	69 (5) 55 (4) 341 (26) 536 (42) 141 (11) 124 (10) 15 (1)
**Knowledge of dengue**	869 (83.4)	189 (76.8)	1058 (82.2)
**Place of birth** Within MAB* Within Santander (but outside of MAB*) *Outside of Santander*	768(74) 133(13) 136(13)	210(85) 24(10) 12(5)	979(76) 158(12) 148(12)
**Self-reported history of dengue disease**^ɸ^	218 (21)	11 (4)	229 (18)
** Diagnosis of dengue by a physician**ᶧ	204 (93.58)ᶧ	10 (90.91)	214 (93.45)
**Yellow fever vaccination**	752 (72)	181 (73)	933 (72)
**Time living in Piedecuesta (years). Median±IQR**	11±17	15±19	12±17
**Location where they spend most of the day** *Family home* *Work* *School* *University* *Other*	45(4) 275(26) 321(31) 98(9) 184(18)	11(5) 87(35) 86(35) 0(0) 31(13)	56(4) 362(28) 407(32) 98(8) 215(17)
**Time spent outside the house daily (hours). Median±IQR**	6±4	6±3	6±4
**Furthest place traveled in the last 6 months** *Didn’t report travel* Within MAB* Within Santander (but outside of MAB*) *Another place outside of Santander (other locations of Colombia)* *Unknow*	312(30) 296(29) 199(19) 228(22) 2(1)	110(45) 89(36) 22(9) 25(10) 0(0)	422(33) 385(30) 221(17) 253(20) 2(1)

*MAB = Metropolitan area of Bucaramanga.

ɸ From children between 2 to 5 years old the response was given by the parents/guardians.

ᶧThis proportion was calculated taking as a denominator the number of participants who “self-reported history of dengue disease”.

On average, 13 households (range 10–22) were enrolled per block in the urban area, and 11 households (range 8–18) in the rural “vereda”. The median number of families per households was one (range 1–5) and the median number of people living in each household was 4 (range 1–20). Households located in urban areas were spread across several socio-economic strata (as defined by the Colombian socio-economic stratification system) but had universal access to basic services such as electricity, water and drainage (Table 2). In contrast, rural households were concentrated in the lowest socio-economic stratum (stratum 1) and had more limited access to drinkable water (46% reported obtaining it from a public tap, river or tank) and sewage (22% had latrines and 78% had septic tanks instead of underground drainage).

**Table 2:** Household characteristics of urban and rural areas. Piedecuesta, 2014.

Characteristics	Urban. n (%)	Rural. n (%)	Overall. n (%)
**Number of houses**	533	91	624
**Socio-economic stratum** *1 (lowest)* *2* *3* *4 (highest)*	19 (4) 155 (29) 318(60) 41 (8)	91 (100)	110(18) 155(25) 318(51) 41(7)
**Type of household** *House* *Apartment* *Multi-housing* *Room and other*	426 (79.9) 84 (15.7) 23 (4.3) 0 (0)	88 (96.7) 0(0) 0 (0) 3 (3.3)	514 (82.3) 84 (13.4) 23 (3.6) 3 (0.4)
**Number of people/household. Median±IQR**	4±2	4±2	4±2
**Number of rooms. Median±IQR**	3±1	2±1	3±1
**Electricity**	533 (100)	90 (98.9)	623 (99.8)
**Basic sanitation (%)**	531 (99.6)	24 (26.3)	555 (88.9)
**Water supply (%)** *Aqueduct* *River*, *well and public tap*	533 (100) 0 (0)	49 (54) 42 (46)	582 (93.2) 42(6.8)
**Type of sewage (%)** *Drainage system* *Septic tank* *Latrine and have no toilet*	533 (100) 0 (0) 0 (0)	0 (0) 71 (78) 20 (22)	533 (85.4) 71 (11.3) 20 (3.3)
**Garbage Collection (%)** *Truck* *Burn* *Bury* *Other way*	532 (99.8) 0 (0) 0 (0) 1 (0.1)	0 (0) 68 (74.7) 6 (6.5) 16 (18.6)	532(85.2) 68 (10.9) 6 (0.9) 19(4)
**Air-conditioning (%)**	7 (1.3)	0(0)	7 (1,12)
**Fan (%)**	310 (58.1)	0 (0)	310 (49.6)
**Periodic fumigation of household (%)**	269 (50.4)	10 (10.9)	279 (44.7)
**Days since last fumigation. Median±IQR.**	8±12	4.5±4	7±12
**Wash laundry tubs. (%)**	490 (91.9)	85 (93.4)	575 (92.1)
**Presence of domestic animals**	296 (55.5)	85 (93.4)	381 (61.1)
**Nets in windows**	18 (3.3)	2 (2.2)	20 (3.2)
**Water containers in residence**	176 (33)	43 (47.2)	219 (35.1)
**Covered containers**	84(47.7)	9(20.9)	93 (42.4)

When asked about behaviors and practices related to vector control, 50% (269/533) of households in the urban area reported periodic fumigating (median 1 time per week) while only 19/91 (10%) households reported this practice in the rural area. Most of the households had laundry tubs (575/624, 92%) but only 512 (89%) reported washing them periodically. Similarly, while 219/624 (35%) of households had at least one large water container, only 93(42%) were properly covered.

### DENV seropositivity

**Fig 3:**
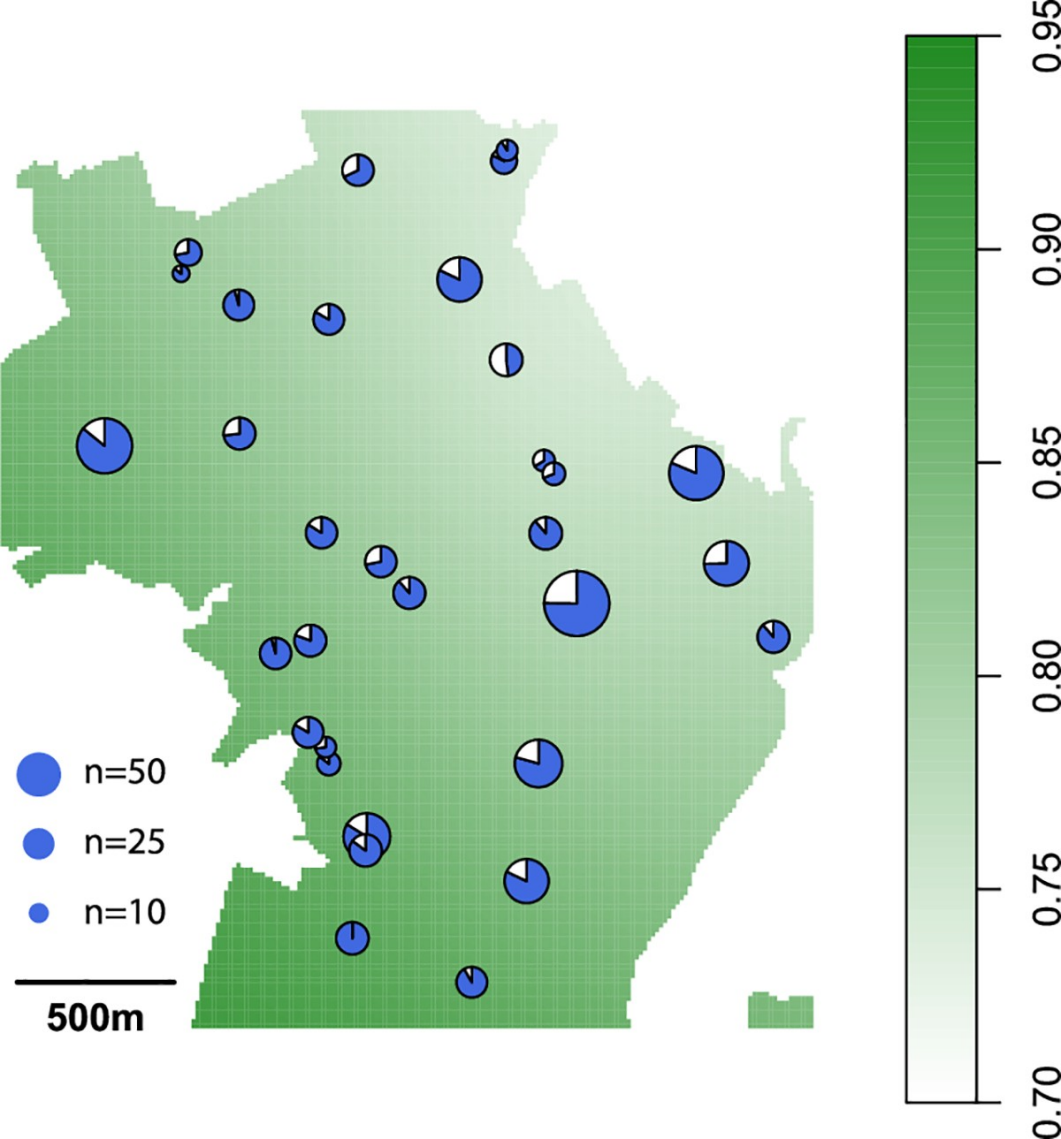
Heatmap showing locations and seropositivity of sampled urban blocks.

Overall, 896/1284 samples (70%, 95% CI = 67% - 71%) tested positive for IgG antibodies against DENV. The seroprevalence was significantly higher among urban households (81%, 95% CI = 78% - 83%) as compared to rural households (21%, 95% CI = 17%- 27%; p = <0.000) which is not unexpected given the difference in elevation of the two settings. We found no evidence of spatial heterogeneity in seropositivity in urban Piedecuesta (Fig 3).

**Fig 4:**
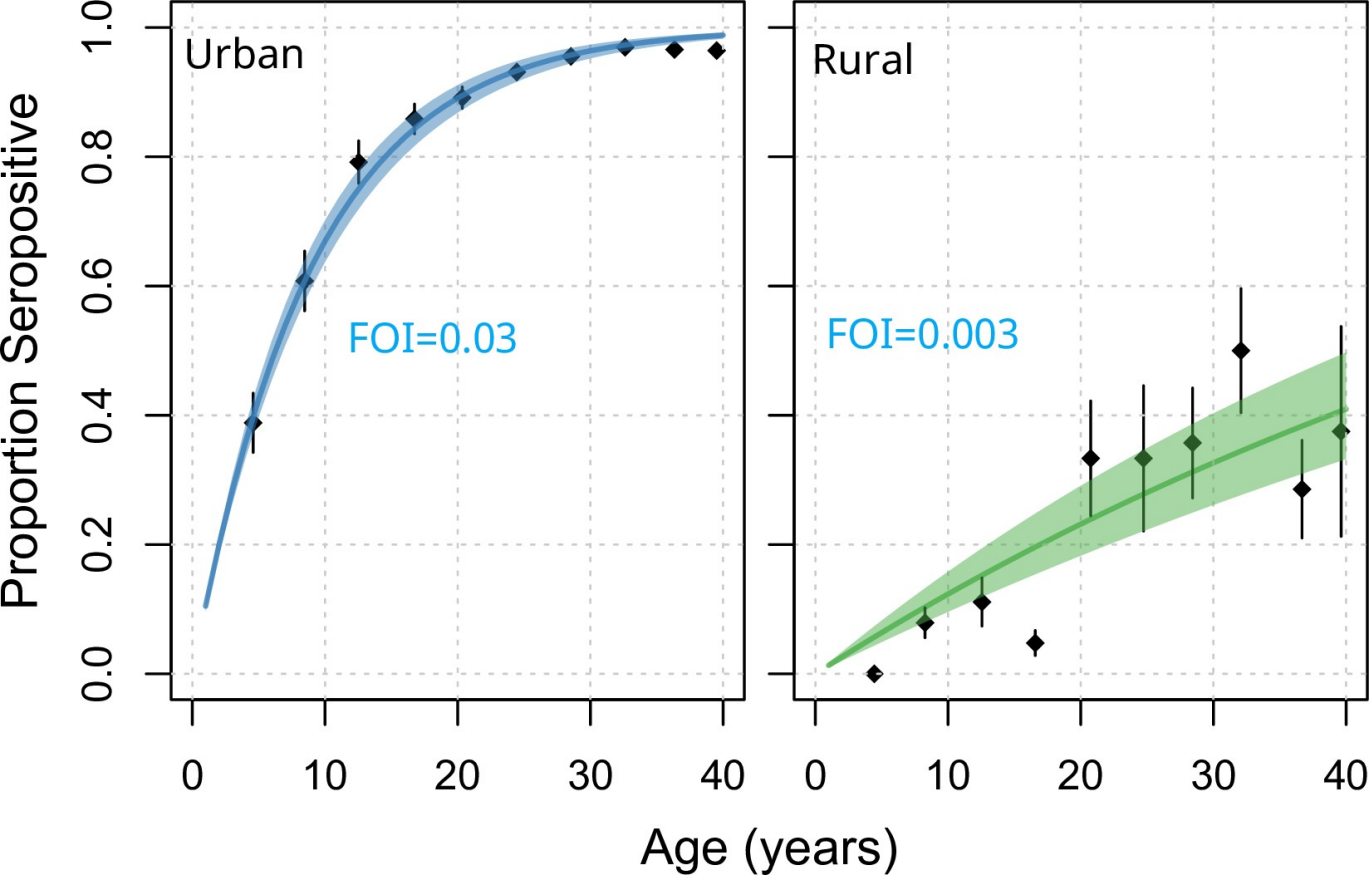
Age specific seroprevalence of DENV in urban (left) and rural (right) Piedecuesta, 2014.

As expected in settings with endemic transmission, age was the main predictor of seropositivity. By fitting catalytic models to the age-stratified data we estimated an average force of infection of 0.030 (95%CI 0.010–0.040) per serotype in urban Piedecuesta and of 0.003 (95% 0.002–0.004) in the rural “vereda” (Fig 4). These forces of infection imply that on average, 12% (95%CI = 11%-13%) of the susceptible individuals living in the urban setting get infected each year by DENV, as compared to only 1.3% of individuals living in the rural area. Based on these transmission intensities, we estimate that the basic reproductive number (R_0_) of DENV is 2.1 (95%CI = 2.0–2.2) in the urban locations and 1.1 (95%CI = 1.0–1.1) in the rural locations.

### Risk factors associated with seropositivity

Among participants who lived in urban Piedecuesta, several individual and household-level variables were associated with seropositivity in unadjusted analyses. Beyond age, these included educational attainment, history of dengue and time living in Piedecuesta (Table 3). However, most of these associations were not significant in adjusted models. The best fitting model included age and history of dengue diagnosed by a physician. Reporting a previous episode of dengue clinically diagnosed by a physician increased the prevalence by a factor of 1.15 (95% CI = 0.98–1.35). We did not find associations between socioeconomic stratum or vector control strategies (eg; fumigation, washing and covering the water tank) and seropositivity. Similarly, we did not find an association between travel to other locations within or outside of Metropolitan area of Bucaramanga neither within nor outside of the Santander department and DENV seropositivity.

**Table 3:** Results of unadjusted Poisson multilevel regression of the association between seropositivity to DENV and several household and individual factors in urban/rural area of Piedecuesta, 2014.

Variable	Unadjusted Prevalence Ratio (95% IC). Urban area (n = 1005)	Unadjusted Prevalence Ratio (95% IC). Rural area (n = 242)
**Individual Characteristics**		
**Age (in years)** z *2–5* *6–15* *16–25* *26–40*	Ref 2.08(1.42–3.06) 2.85(1.96–4.13) 3.00(2.07–4.33)	Ref. 1.73(0.20–14.43) 4.83(0.62–37.38) 8.19(1.12–59.7)
**Gender- Male**	1 (0.78–1.03)	0.68 (0.38–1.19)
**Place of birth** Within MAB* Within Santander (but outside of MAB*) *Outside of Santander(other locations of Colombia)*	Ref. 1.07(0.87–1.31) 1.03(0.84–1.27)	Ref. 1.29(0.54–3.05) 3.2(1.47–7.35)
**Education** *None* *Preschool* *Primary* *Secondary* *Technical* *Undergraduate* *Postgraduate*	Ref 1.97 (1.19–3.27) 2.50 (1.53–4.06) 2.65 (1.59–4.40) 2.61 (1.56–4.35) 2.82 (1.37–5.8) 0.98 (0.49–1.94)	Ref 2.9(0.40–21.88)ᶧ 4.1(0.56–30.9)ᶧᶧ
**Knowledge of dengue**	1.6 (1.30–2.04)	2.2(0.96–5.2)
**Self-reported history of dengue disease**	1.21 (1.03–1.42)	1.2(0.39–4)
**Diagnosis of dengue by a physician**	1.22 (1.03–1.43)	1.3(0.43–4.4)
**Yellow fever vaccination**	1 (0.99–1.02)	1(0.93–1.09)
**Time living in Piedecuesta (years)**	1,01 (1,00; 1,01)	1.02(1–1.04)
**Location where they spend most of the day.** *None* *Family home* *Work* *School* *University* *Other*	Ref. 1.06(0.73–1.55) 1.13(0.89–1.44) 0.77(0.60–1.00) 1.09(0.81–1.46) 0.99(0.76–1.29)	Ref. 0.58(0.22–1.53) 0.13(0.03–0.45) 0.95(0.33–2.67) 0.51(0.16–1.61)
**Time spent outside the house daily (hours)**	1 (0.99–1.02)	0.95(0.87–1.04)
**Furthest place traveled in the last 6 months** *Didn’t report travel* Within MAB* Within Santander (but outside of MAB*) *Outside of Santander (other locations of Colombia)* *Unknow*	Ref. 1.06(0.89–1.27) 1.08(0.89–1.32) 1.06(0.87–1.28) 1.29(0.18–9.20)	Ref. 2.3(1.1–4.7) 4.5(2–10) 2.7(1.08–7)
**Household Characteristics**		
**Number of people**	0.98(0.96–1.01)	0.94(0.82–1.08)
**Socioeconomic stratum**		
*1* *2* *3* *4*	Ref. 0.89(0.64–1.2) 0.85(0.62–1.17) 0.87(0.56–1.33)	
**Household has a fan**	0.96(0.84–1.11)	
**Periodic fumigation of household**	1.01(0.88–1.15)	1.07(0.48–2.3)
**Presence of domestic animals**	1.07(0.93–1.23)	0.45(0.22–0.90)
**Wash laundry tubs**		2.4(0.58–9.95)

*MAB = Metropolitan area of Bucaramanga.

ᶧ Preschool or primary

ᶧᶧ Other (secondary or technical).

While our study was not powered to detect associations in the rural setting alone, we also fit models to evaluate associations with individual and household level covariates (Table 4). People who were born outside of the Santander department were more likely to be seropositive (aPR = 2.77, 95%CI = 1.20–6.37). Similarly, people who reported traveling outside of Piedecuesta, but within Santander, were also at higher risk (aPR = 3.60, 95%CI 1.54–8.42). In addition, the presence of domestic animals was associated with significantly decreased risk of being seropositive (aPR = 0.36, 95% CI = 0.17–0.74).

**Table 4:** Results of multivariate Poisson multilevel regression of the association between seropositivity to DENV and several household and individual factors in urban/rural area of Piedecuesta, 2014.

Variable	Adjusted Prevalence Ratio (95% IC). Urban area (n = 1005)	Adjusted Prevalence Ratio (95% CI). Rural área(n = 242)
**Individual Characteristics**		
**Age (in years)** *2–5* *6–15* *16–25* *26–40*	Ref 2.06(1.40–3.03) 2.79(1.92–4.06) 2.95(2.04–4.26)	Ref. 1.83(0.21–15.34) 4.26(0.53–33.75) 6.96(0.92–52.40)
**Place of birth** Within MAB* Within Santander (but outside of MAB*) *Outside of Santander(other locations of Colombia)*		Ref. 0.91(0.37–2.25) 2.77(1.20–6.37)
**Diagnosis of dengue by a physician**	1.15(0.98–1.35)	
**Furthest place traveled in the last 6 months** *Didn’t report travel* Within MAB* Within Santander (but outside of MAB*) *Another place outside of Santander (other locations of Colombia)*		Ref. 1.68(0.81–3.47) 3.60(1.54–8.42) 1.90(0.71–5.06)
**Household characteristics**		
**Presence of domestic animals**		0.36(0.17–0.74)

*MAB = Metropolitan area of Bucaramanga

## Discussion

Dengue is a public health problem in Colombia and yet few studies have characterized the extent of DENV transmission in endemic areas of the country. Here, we present the results of a population-based serosurvery conducted among the general population of Piedecuesta Santander, including urban and rural areas.

Our findings are consistent with long-term endemic circulation of DENV in the urban area of Piedecuesta. We estimate that 81% of individuals aged 2–40 years been infected by DENV at least once, and that on average, 12% of the susceptible population gets infected each year. This high transmission intensity is consistent with that reported in other endemic areas of the world such as Thailand, Nicaragua, Brazil and Mexico[20–21]. In contrast to other studies that have found strong associations between socio-economic [22–25], demographic [26–32], and behavioral [33–37] variables and DENV transmission, our findings are consistent with a spatially homogeneous transmission and no evidence of any housing or environmental factors related to prior exposure to DENV.

Compared to the high seropositivity among participants living in urban Piedecuesta, only 21% of individuals in rural area were found to be DENV seropositive despite living only 10km away from the urban center. This is not unexpected given that the rural vereda is located over 2.100m above sea level, an altitude that is known to be unsuitable for *Aedes aegypti* [38]. However, the possibility of finding the vector above 1800m is not ruled out according to previous studies in other areas of Colombia[39–40]. While we estimated that 1.2% of susceptible individuals living in rural Piedecuesta get infected each year, it is likely that these infections occurred when individuals travel to nearby endemic areas. This hypothesis is supported by the positive association between reporting traveling to other areas of the Santander and DENV seropositivity observed only among individuals from rural Piedecuesta. However, entomological studies would be required before confirming that all of these infections are travel associated and that there is no DENV transmission within this rural “vereda”. Interestingly, we found a lower seropositivity related to owning domestic animals, although only in rural houses.

While the large difference in seropositivity between urban and rural Piedecuesta is not unexpected, it highlights the large heterogeneity that may exist in DENV transmission, even at fine spatial scales. This finding suggests that, in many settings, decisions on where to target interventions such as vaccination and vector-control will need to be made beyond the second administrative level. While in urban Piedecuesta, children aged 12 years or older would’ve met the initial recommendation by the WHO (seropositivity >70%) and would have been candidates for vaccination, vaccination would have been contraindicated in the rural area, even among adults. These findings support the new recommendation by the WHO of using pre-vaccination screening to ascertain whether an individual has been infected by DENV in the past[8]. Such recommendation will be essential to minimize the chances of vaccinating seronegative individuals in places where transmission is highly heterogeneous.

This study has several limitations. While the sample obtained from the urban population was probabilistic, and is likely to represent the underlying population of urban Piedecuesta, rural samples were obtained from a single rural vereda that was chosen based on its accessibility for the study team. Therefore, the findings from this rural location are probably not generalizable to the all of the rural population from this municipality. Sampling from additional rural “veredas”, and in particular from low-altitude rural areas, would be necessary to ascertain whether there are differences in the transmission intensity of DENV between rural and urban settings.

The serological assay used only provides information on whether a person has been previously infected, but does not provide any information about serotypes. Further testing of these samples using plaque reduction neutralization assays would be desirable to characterize whether immunity is monotypic (against a single serotype) or multitypic and to get an idea of what serotypes have circulated in this region. However based on data from national surveillance and from AEDES Network projects (www.redaedes.org), DENV-1 to -4 serotypes have been isolated from this region in the past 19 years and therefore it is likely that a large proportion of participants has been infected by more than one serotype[11–41–42].

Population based seroprevalence studies are needed from other municipalities of Colombia and the American continent to know if the transmission dynamics of DENV and the factors associated seropositivity differ between regions. This information will be fundamental to consolidate the Integrated Management Strategy for DENV prevention that is being developed in Colombia and guide targeting of interventions.

## Supporting information

S1 Table Urban area: Results of multivariate Poisson multilevel regression (n = 1005). (PDF)

S2 Table Rural area: Results of multivariate Poisson multilevel regression (n = 242). (PDF)
