# Supplementary material for: Heterogeneity of dengue transmission in an endemic area of Colombia
Source: PLoS Negl Trop Dis. 2020 Sep 14;14(9):e0008122. doi: 10.1371/journal.pntd.0008122 (PMC7571714; doi:10.1371/journal.pntd.0008122)
Supplement: S1 Table — (PDF) [file pntd.0008122.s001.pdf]

S1 Table. Urban area: Results of multivariate Poisson multilevel regression (n=1005)

Variables	Model 0<br>PR (95%IC)	Model 1<br>PR (95%IC)	Model 2<br>PR (95%IC)	Model 3<br>PR (95%IC)	Model 4 - Final<br>PR (95%IC)
Constant	0.81(0.75-0.87)	0.31(0.22-0.45)	0.32(0.22-0.46)	0.31(0.22-0.44)	0.31(0.22-0.44)
**Individual Characteristics**					
**Age of groups (years)**					
2 -5		Ref.	Ref.	Ref.	Ref
6-15		2.08(1.42-3.06)	2.08(1.41-3.05)	2.06(1.40-3.03)	2.06(1.40-3.03)
16-25		2.85(1.96-4.13)	2.84(1.95-4.12)	2.79(1.92-4.05)	2.79(1.92-4.06)
26-40		3(2.07-4.33)	2.98(2.06-4.31)	2.95(2.04-4.26)	2.95(2.04-4.26)
**Gender- Male**			0.97(0.84-1.13)		
**History of dengue (self-report)**				1.14(0.97-1.34)	
**Diagnosis of dengue by a Physician**					1.15(0.98-1.35)
**ICC**	6.68e-09	8.34e-09	7.40e-09	2.88e-08	2.84-08
**AIC**	1976	1925	1927	1924	1924
