# Supplementary material for: Heterogeneity of dengue transmission in an endemic area of Colombia
Source: PLoS Negl Trop Dis. 2020 Sep 14;14(9):e0008122. doi: 10.1371/journal.pntd.0008122 (PMC7571714; doi:10.1371/journal.pntd.0008122)
Supplement: S2 Table — (PDF) [file pntd.0008122.s002.pdf]

S2 Table. Rural area: Results of multivariate Poisson multilevel regression (n=242)

**Variables**	**Model 0**<br>**PR (95%IC)**	**Model 1**<br>**PR (95%IC)**	**Model 2**<br>**PR (95%IC)**	**Model 3**<br>**PR (95%IC)**	**Model 4**<br>**PR (95%IC)**	**Model 5**<br>**PR (95%IC)**
Constant	0.21(0.16-0.28)	0.04(0.00-0.32)	0.05(0.00-0.37)	0.04(0.00-0.30)	0.033(0.004-0.24)	0.037(0.00-0.26)
**Individual Characteristics**						
**Age of group (years)**						
2 -5		Ref	Ref.	Ref.	Ref.	Ref.
6-15		1.73(0.20-14.43)	1.73(0.20-14.37)	1.81(0.21-15.07)	1.72(0.20-14.41)	1.58(0.19-13.23)
16-25		4.83(0.62-37.48)	4.72(0.60-36.59)	4.89(0.63-37.9)	3.74(0.47-29.52)	3.50(0.44-27.71)
26-40		8.19(1.12-59.79)	8(1.09-58.46)	7.94(1.08-58.15)	6.29(0.84-46.91)[†]	6.16(0.82-46.02)
**Gender- Male**			0.73(0.41-1.28)			
**Place where was born**						
Within Area Metropolitana de Bucaramanga				Ref.	Ref.	
Within Santander				0.93(0.39-2.21)	0.76(0.31-1.86)	
Outside of Santander				2.39(1.06-5.39)	2.66(1.15-6.12)	
**Furthest place traveled in the last 6 months**						
Didn't report travel					Ref.	Ref.
Within Area Metropolitana de Bucaramanga					1.60(0.78-3.29)	1.64(0.80-3.37)
Within Santander					3.71(1.58-8.69)	3.26(1.41-7.54)
Another place outside of Santander					1.60(0.61-4.19)	1.78(0.68-4.62)
**Household Characteristics**						
**Have domestic animals**						
**Wash laundry tubs**						
**ICC**	9.18e-07	2.10e-07	2.04e-07	1.69e-07	7.32e-08	1.94e-07
**AIC**	271	255	256	255	252	253

(Continued table)

	Model 6 PR (95%IC	Model 7 PR (95%IC	Model 8 PR (95%IC	Model 7-Final PR (95%IC)
Constant	0.07(0.01-0.57)	0.036(0.00-0.40)	0.024(0.00-0.30)	0.06(0.00-0.52)
**Individual Characteristics**				
**Age of group (years)**				
2 -5	Ref.	Ref.	Ref.	Ref.
6-15	1.71(0.20-14-42)	1.64(0.19-13.84)	1.81(0.21-15.22)	1.83(0.21-15.34)
16-25	4.23(0.52-33.87)	4(0.49-32.1)	4.11(0.51-32.52)	4.26(0.53-33.75)
26-40	7.07(0.93-53.5)	6.93(0.91-52.4)	7.02(0.93-52.74)	6.96(0.92-52.40)
**Gender- Male**				
**Place where was born**				
Within Area Metropolitana de Bucaramanga			Ref.	Ref.
Within Santander			0.92(0.37-2.27)	0.91(0.37-2.25)
Outside of Santander			3.16(1.37-7.31)	2.77(1.20-6.37)
**Furthest place traveled in the last 6 months**				
Didn't report travel	Ref.	Ref.	Ref.	Ref.
Within Area Metropolitana de	1.66(0.81-2.41)	1.62(0.79-3.31)	1.62(0.79-3.34)	1.68(0.81-3.47)
Bucaramanga			3.42(1.46-8.00)	
Within Santander	3.20(1.39-7.34)	2.99(1.30-6.89)		3.60(1.54-8.42)
Another place outside of Santander	2.08(0.79-5.47)	2.14(0.81-5.60)	2.22(0.83-5.90)	1.90(0.71-5.06)
**Household Characteristics**				
**Have domestic animals**	0.36(0.17-0.73)	0.36(0.18-0.74)	0.36(0.17-0.74)	0.36(0.17-0.74)
**Wash laundry tubs**		2.30(0.55-9.63)	2.96(0.67-12.9)	
**ICC**	1.56e-07	1.00e-09	7.57e-08	1.97e-07
**AIC**	249	247	248	248
